# Establishing the cascade of care for patients with tuberculous meningitis

**DOI:** 10.12688/wellcomeopenres.15515.2

**Published:** 2020-01-30

**Authors:** Darma Imran, Philip C. Hill, Jacob McKnight, Reinout van Crevel, Tuberculous Meningitis International Research Consortium, Rob E. Aarnoutse, Rob E. Aarnoutse, Suzanne T. B. Anderson, Suzanne T. B. Anderson, Nathan C. Bahr, Nguyen D. Bang, David R. Boulware, Tom Boyles, Lindsey H. M. te Brake, Satish Chandra, Felicia C. Chow, Fiona V. Cresswell, Reinout van Crevel, Angharad G. Davis, Sofiati Dian, Joseph Donovan, Kelly E. Dooley, Anthony Figaji, A. Rizal Ganiem, Ravindra Kumar Garg, Diana M. Gibb, Raph L. Hamers, Nguyen T. T. Hiep, Darma Imran, Akhmad Imron, Sanjay K. Jain, Sunil K. Jain, Byramee Jeejeebhoy, Jayantee Kalita, Rashmi Kumar, Vinod Kumar, Arjan van Laarhoven, Rachel P-J. Lai, Abi Manesh, Suzaan Marais, Vidya Mave, Graeme Meintjes, David B. Meya, Usha K. Misra, Manish Modi, Alvaro A. Ordonez, Nguyen H. Phu, Sunil Pradhan, Kameshwar Prasad, Alize M. Proust, Lalita Ramakrishnan, Ursula Rohlwink, Rovina Ruslami, Johannes F. Schoeman, James A. Seddon, Kusum Sharma, Omar Siddiqi, Regan S. Solomons, Nguyen T. T. Thuong, Guy E. Thwaites, Ronald van Toorn, Elizabeth W. Tucker, Sean A. Wasserman, Robert J. Wilkinson

**Affiliations:** 1 Department of Neurology, Cipto Mangunkusumo Hospital, Faculty of Medicine University of Indonesia, Jakarta, Indonesia; 2 Center for International Health, University of Otago, Dunedin, New Zealand; 3 Oxford Health System Collaboration, Oxford University, Oxford, UK; 4 Centre for Tropical Medicine and Global Health, Nuffield Department of Medicine, University of Oxford, Oxford, UK; 5 Internal Medicine and Radboud Center for Infectious Diseases, Radboud University Nijmegen Medical Centre, Nijmegen, The Netherlands

**Keywords:** tuberculosis, meningitis, cascade of care, patient pathway analysis, health systems

## Abstract

Meningitis is a relatively rare form of tuberculosis, but it carries a high mortality rate, reaching 50% in some settings, with higher rates among patients with HIV co-infection and those with drug-resistant disease. Most studies of tuberculosis meningitis (TBM) tend to focus on better diagnosis, drug treatment and supportive care for patients in hospital. However, there is significant variability in mortality between settings, which may be due to specific variation in the availability and quality of health care services, both prior to, during, and after hospitalization. Such variations have not been studied thoroughly, and we therefore present a theoretical framework that may help to identify where efforts should be focused in providing optimal services for TBM patients. As a first step, we propose an adjusted cascade of care for TBM and patient pathway studies that might help identify factors that account for losses and delays across the cascade. Many of the possible gaps in the TBM cascade are related to health systems factors; we have selected nine domains and provide relevant examples of systems factors for TBM for each of these domains that could be the basis for a health needs assessment to address such gaps. Finally, we suggest some immediate action that could be taken to help make improvements in services. Our theoretical framework will hopefully lead to more health system research and improved care for patients suffering from this most dangerous form of tuberculosis.

## Introduction

Tuberculosis (TB) is a global health emergency, leading to more than 10 million new cases and an estimated 1.6 million deaths in 2017 ^1^. Tuberculous meningitis (TBM) only makes up a small proportion of TB cases (1–2%, probably more in human immunodeficiency [HIV]-endemic settings) but disproportionally contributes to TB-associated mortality ^2^. Up to 50% of TBM patients in published studies die, and mortality is substantially higher among those with drug-resistant TB or HIV co-infection ^2^. An unknown proportion of TBM patients even die before diagnosis is made or treatment is started. Moreover, studies with longer follow-up show that TBM patients remain at increased risk of dying after completion of TB treatment ^3, 4^. Finally, among those patients who survive, some will have permanent disability.

Mortality of TBM shows high variability between settings. This may be due to differences in disease severity, prevalence of HIV co-infection and drug resistance, but could also be explained by specific variation in availability and quality of health care services (health system factors). The Lancet Global Health Commission on high quality health systems, published in 2018, estimated that 50% of TB deaths result from poor-quality care ^5^. This figure might even be higher for TBM, as its diagnosis and treatment are complex and technically demanding, requiring advanced diagnostics and specialized care which are often either absent or suboptimal in low-resource settings.

Research aimed at improving outcome on TBM mainly focuses on better diagnosis ^6– 8^, drug treatment ^2, 9, 10^ and supportive care for patients in hospital ^11^, rather than on the patient’s journey from the moment he or she develops symptoms until no further care is needed. In the absence of empirical data, we aim to provide a theoretical framework that may help identify barriers and challenges in providing optimal care for TBM patients, by combining cascade of care, patient pathway and health needs analyses. This framework will hopefully lead to more health system research to assess and improve the quality of care for patients with this most dramatic manifestation of TB.

## Cascade of care analysis

The outcome of TBM patients depends on the care they receive, which is a complex process, comprising a cascade of essential steps, with each step unable individually to guarantee a good outcome. The TB care cascade represents a normative model, based on the International Standards for Tuberculosis Care, which defines the proper stages of high-quality TB treatment ^12^. In its most simple form, it starts with the number of TB patients (the first step in the cascade); followed by the number of patients that accesses TB services or testing; then the number diagnosed with TB; started on treatment; and then finally, the number who successfully complete treatment ^13^. Secondary cascades can be drawn for subgroups of patients, for instance when drug-resistance is diagnosed ^14^. TB programs can use cascade of care analysis to further assess their performance in key processes and, after identifying the underlying reasons for the losses found, to prioritize areas for focused improvement ^12^.

**Figure 1.**
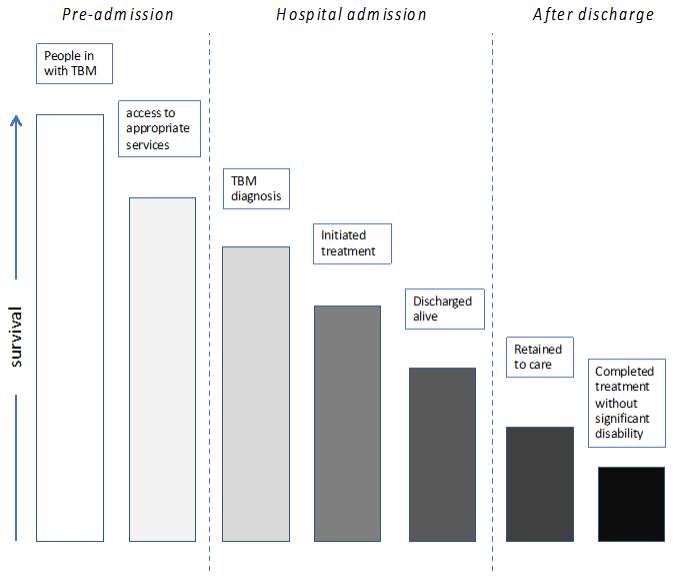
Hypothetical cascade of care for tuberculosis meningitis (TBM). Hypothetical cascade for TBM, that is not based on universal guidelines or empiric data. This simplified cascade does not take time between steps into consideration. Additional cascades of care can be drafted for patients with HIV co-infection, drug-resistant TBM, or other needs such as critical care or rehabilitation.

To our knowledge, no assessment of the cascade of care have been conducted for TBM. We propose that a theoretical cascade might comprise of the numbers in sequence: TBM patients in a particular community (something that will be very hard to establish); those accessing a health facility able to diagnose TBM; those diagnosed as TBM; those started on treatment; those discharged alive; those retained to care after discharge; and those completing treatment without significant disability ( Figure 1). It should be noted that this cascade is not based on universal international guidelines but rather represents an ‘ideal scenario’ that in high-burden settings may only exist in some centers, with qualified or experienced professionals and appropriate services. It should also be noted that secondary cascades can be drafted for patients with drug-resistant TBM, with HIV co-infection, or with complications requiring critical care, neurosurgical interventions, rehabilitation or appropriate support because of neurological disability etcetera.

The first loss in the cascade is related to access of patients to health services that are appropriately equipped for TBM diagnosis. The second loss across the cascade is related to diagnosis of TBM. Even when patients reach appropriate services, doctors may fail to consider or diagnose TBM. Awareness among health care workers to think about brain infections and tuberculosis is needed, and an appropriate diagnostic workup has to be done, including brain imaging and analysis of cerebrospinal fluid (CSF) obtained through a lumbar puncture (LP). Unfortunately, no single diagnostic CSF can rule out TBM. Also, there may be contraindications for doing LP, or patients or their family may refuse LP ^15^.

The third loss is related to treatment. After a presumptive or confirmed diagnosis of TBM is made, treatment should be started immediately, especially for critically ill patients. Besides timely antimycobacterial treatment, critically ill TBM patients require corticosteroids ^16^, optimal supportive or neurocritical care ^11^, and sometimes neurosurgical management treatment ^17^. Patients also need to be monitored closely. Treatment is made up of different components, which creates opportunities for multiple possible gaps between these different elements.

The fourth loss is related to the period after hospitalization. Patients that survive the initial phase and recover will be discharged, mostly after weeks of hospitalization. At this point, patients still need to continue antimicrobial treatment for months, but patients may skip dosages, stop taking medication all together, or be lost to follow-up during ambulatory treatment. Patients with disabilities may need some form of rehabilitation or support, and this may not be available or may not be offered. Furthermore, incomplete drug intake, or lack of follow-up or rehabilitation may contribute to the fact that mortality remains elevated until years after diagnosis of TBM ^3, 4^.

HIV co-infection, which doubles mortality of TBM and adds to the complexity of care for TBM patients ^18^, has its own cascade of care. Patients with an untreated HIV infection have to start antiretroviral treatment (ART) several weeks after initiation of TB drugs but this may not happen, or compliance or treatment monitoring may be suboptimal, or patients may stop taking drugs or be lost to follow-up from HIV care. Similar to HIV co-infection, drug-resistance among TBM patients worsens outcome ^19^ and has its own parallel cascade of diagnosis and treatment. 

Importantly, traditional cascades only measure loss over a cascade, but some studies have also measured the time between steps ^14^. This is very important for TBM, which can be rapidly progressive if no diagnosis is made or treatment started. Time between onset of symptoms and access to appropriate services, presentation and LP, and diagnosis and antibiotic treatment could be prioritized for measurement.

## Patient pathway analysis

Patient pathway studies are used to identify factors that account for losses across a cascade of care. Patient-pathway analyses assess the alignment of health systems’ infrastructure (e.g. diagnostic, referral and treatment capacity) with patients’ care-seeking behavior ^20^. Patient pathways of TBM patients are probably highly variable. Even in relatively well-organized health systems, with good access to healthcare, the trajectory of TBM patients can be short and tragic. As TBM usually has a subacute or non-specific clinical presentation, similar to pulmonary TB ^12^, many patients may pay multiple visits to different health practitioners, and in some patients eventually a diagnosis is made, while in others it is not. Similarly, some patients may be started on appropriate treatment and be retained to care but others not. Importantly, for some patients a successful pathway to diagnosis and treatment may be short, while in others there may be significant delays leading to unnecessary morbidity and mortality.

Patient pathway studies might help identify factors that account for losses and delays across the cascade of care for TBM, the first being access to appropriate health services. The initial presentation of TBM is non-specific, and its diagnostic work-up (different from other forms of TB) can only be done at secondary or tertiary hospitals. Therefore, the first challenge for a patient developing TBM is to reach such specialized health services in time. Patients (and their family members) may not consider the possibility of life-threating brain infection, or may not have access to specialized services. For pulmonary TB, it is estimated that the majority of patients begin seeking care in informal (e.g. traditional or homeopathic doctors, and pharmacists) and private doctors ^5^. This leads to considerable and unnecessary delays and costs to patients. For TBM and other forms of extrapulmonary TB, the situation may be even more difficult; in a recent study in India, only 12% of patients with extrapulmonary TB first sought medical advice, most patients visited three or more clinics, and the average delay to reaching appropriate services was two months ^21^. Even when appropriate services are reached, some patients may need repeated visits to such services, and the time it takes until a diagnosis is reached and treatment is started may vary considerably. Similarly, pathways may vary after hospital discharge.

## Health systems factors

Many of the possible gaps in diagnosis and treatment of TBM are related to health systems factors. Health systems factors, such as the availability of the right facilities or workforce, health information, guidelines, drugs, financing, and organization of the healthy system can explain the wide variability in patient pathways, losses across the ‘cascade of care’ and delays across different settings and countries, and thereby the variable mortality of TBM.

Even though health systems are highly context-specific, they share certain characteristics that are essential for delivering good care. The World Health Organization (WHO) has proposed a framework with six ‘building blocks’ of health systems ^22^. This WHO framework was adjusted to evaluate health systems factors for management of multi-drug resistant (MDR) TB ^23^. We used the nine domains of that study and our experience in Indonesia to illustrate health systems factors that may be relevant for TBM ( Table 1).

**Table 1.** Theoretical framework of health systems factors that are likely to be relevant for tuberculosis meningitis (TBM).

Domain/parameter *	Factors possibly relevant for TBM in Indonesia **
Facilities and specialists	There is often a lack of trained doctors or other health staff with knowledge of neuro-infections.
	Many facilities lack high-level care, necessary laboratory tests and neuroimaging.
Incidence and outcome data	No programmatic data are collected for TBM incidence and outcome to help adjust policy or service delivery.
Available guidance and protocols	There is a national guideline for TBM, but translation of specific care components to patient management protocols is lacking.
Health systems financing	Universal health insurance does not cover cost needed for neuroimaging, critical care and rehabilitation.
	TBM likely inflicts catastrophic costs to patients and their families.
Health and social system organization	Efficiency of referral from primary/secondary care level is often difficult and slow.
	Follow-up of ‘no shows’ (e.g. by social workers) after discharge is not routine.
	Rehabilitation for neurological sequelae is hardly available in Indonesia.
Health systems regulation	Regulation related to health insurance often prohibits patient referral to specific (tertiary) care facilities.
Uninterrupted drug supply	Intravenous antimycobacterial drugs that may be helpful in unconscious patients are unavailable.
Public health information	Information related to TBM targeted to professionals or the general public is frequently lacking.
Behavior and attitude of health care workers (HCW)	HCW can feel ‘hopeless’ in light of the severity of TBM, or may stigmatize patients, especially when HIV co-infected.

* Adjusted from 23. ** Based on the authors’ personal impression or experience ^24^, and not on systematic studies. The situation may be similar for many high-burden countries, but systematic studies are lacking.

The situation may be different in different settings, and as a first step, similar as was done for management of MDR-TB, the conceptual framework could be tailored further for TBM, and questionnaires could be circulated to physicians involved in TBM management to help identify and possibly amend health systems issues. Single studies have evaluated some of the factors in Table 1. For instance, with respect to facilities necessary for TBM diagnosis, in a survey among Indonesian neurologists, only 74% mentioned that they had access to routine CSF analysis (cells, protein, glucose), and only 26% and 34% had access to CSF molecular testing and culture, respectively, to confirm TBM ^24^.

## A health needs assessment to quantify gaps in care

A health needs assessment framework takes the measurement of indicators of performance across system parameters and quantifies the gaps in care, which vary between settings, against an ‘ideal’ system. It then considers, using pre-determined criteria, different options to fill each gap. We have previously used a public health framework to identify gaps between current and ideal practice for management of child-case TB contacts ^25^. Based on such an assessment, interventions that will help most in a particular setting can be selected; this concept is now examined for management of latent TB in a multi-country cluster-randomized clinical trial ^26^. Based on Table 1, performance indicators can be identified for TBM, such as: the availability of a neurologist trained in neuro-infections at a facility; the availability of appropriate diagnostics; and management protocols. Sometimes, assessment of policy-practice gaps results in simple action, like advocacy to hospital management for making certain laboratory tests available, like Xpert MTB/RIF or cryptococcal antigen testing on CSF (Darma Imran, personal experience). Or, if gaps are identified in ambulatory care after hospital discharge, development of a simple discharge protocol might help to ensure that medication is continued correctly, that ambulatory follow-up is ensured, and that patients and family are adequately counselled about compliance and possible disease complications that may occur later, such as toxicity or paradoxical worsening.

## Possible next actions

Establishing the cascade of care for TBM, conducting a patient pathway analysis, and further study of health systems factors could help identify priority areas for further action to improve care and outcomes for TBM patients. The health needs assessment as described in the previous paragraph is likely to reveal a lack of knowledge and awareness about TBM and other brain infections among the general public, contributing to late presentation. Immediate action might therefore include some kind of community engagement to stimulate earlier presentation. Such engagement should probably also involve community clinics and doctors; in a cohort study in Jakarta, two thirds of patients presenting at a tertiary hospital with a possible central nervous system infection were self-referrals who had visited other health providers ^27^.

Based on our experience, further study is also likely to identify significant gaps in diagnosis and treatment once patients reach tertiary facilities. Different interventions might be needed to address these gaps, but even without a systematic assessment, development and socialization of management protocols seems a rational thing to do. This might for instance include simple guidance and socialization of indications, contraindications and optimal yield from LPs in high-endemic settings.

Two things may complicate efforts to improve the outcome of TBM using our proposed approach focusing on the cascade of care and quality of services. First, brain infections including TBM are relatively rare, and as such – although they have a huge and often devastating impact on individual patients and their families – have less priority for policy makers. For instance, for national programs, TBM has no priority as it does not pose a public health risk in terms of transmission.

Second, care for TBM is very complex. It has previously been shown that it is not individual factors that make or break a technology implementation effort but the interaction between these individual factors. For interventions (like standard care for TBM) or innovations (new tools for TBM management), the more complex a setting in which it is introduced (like a busy emergency room in a high-burden setting), the less likely it is to be successfully adopted, scaled up, spread, and sustained ^28, 29^. Implementing new or complex medical care beyond individual facilities can be very difficult, especially in low-resource settings. In an effort to address this challenge, a recent publication describes the use of the ‘nonadaptation, abandonment, scale-up, spread and sustainability’ (NASSS) framework for complex interventions ^30^. This framework, based on an extensive literature review of previous technology implementation frameworks and empirical study, helps raise challenges, classified as ‘simple’, ‘complicated’ or ‘complex’. Care for TBM is definitely not simple, but this framework might help address some of the interacting challenges related to the adoption scale-up, distant spread, and long-term sustainability of care for TBM patients.

## Conclusion

Mortality of TBM is highly variable between settings and this may be due to specific variation in the availability and quality of health care services, both prior to, during and after hospitalization. To address this knowledge gap, we have proposed a cascade of care and patient pathway analysis to address factors underlying gaps and delays in this cascade, and nine health systems domains that we think are relevant for TBM and that could help design a structured health needs assessment to address gaps in care. This theoretical framework will hopefully lead to more health system research and improved care for patients suffering from TBM as the most dramatic manifestation of TB.

## Data availability

No data are associated with this article.
