# Supplementary material for: Establishing the cascade of care for patients with tuberculous meningitis
Source: Wellcome Open Res. 2020 Jan 30;4:177. Originally published 2019 Nov 13. [Version 2] doi: 10.12688/wellcomeopenres.15515.2 (PMC7008603; doi:10.12688/wellcomeopenres.15515.2)
Supplement: Peer review history

# Peer review: Reviewer response for version 2

**DOI:** 10.21956/wellcomeopenres.17229.r37759

**Published:** 2020-02-07

**Authors:** Lutgarde Lynen

**Affiliations:** 1 Department of Clinical Sciences, Institute of Tropical Medicine, Antwerp, Belgium

I agree with the revisions. Thanks, It can be indexed in its revised form.

I confirm that I have read this submission and believe that I have an appropriate level of expertise to confirm that it is of an acceptable scientific standard.

# Peer review: Reviewer response for version 1

**DOI:** 10.21956/wellcomeopenres.16982.r37565

**Published:** 2020-01-27

**Authors:** Lutgarde Lynen

**Affiliations:** 1 Department of Clinical Sciences, Institute of Tropical Medicine, Antwerp, Belgium

Page 3 second column:

1. The cascade of care for TB meningitis, a hypothetical framework.The main problem here is how to determine the number of patients with TB meningitis in the community, your denominator? We know how many are diagnosed, but we have no idea on how many are unnoticed. How would you estimate the denominator?
2. You mention that a problem of the cascade is that it only applies to an ideal scenario, and that it may not be available in all settings. I do not entirely agree, you may also discuss the appropriate level of care adapted to the available resources.
3. In the second loss of the cascade I would also add the **awareness** among health care workers to think about tuberculosis, as the initial step in the diagnostic process. Many patients with grade 1 BMRC severity of TBM (GCS normal and no focal signs) may never get a diagnostic work-up started.
4. And at the end (part of the second loss), failure to make a diagnosis of **presumptive TB meningitis**, because all tests are negative. No test is sensitive enough to rule out TB meningitis, and clear guidance should be given to health care workers when to go for a presumptive TB diagnosis and treatment. In HIV-TB co-infected patients, the guidelines to improve the diagnosis of smear-negative TB in HIV prevalent settings (WHO 2006) led to a reduction in diagnostic delay and treatment delay ^1^^,^^2^.

The required probability of TBM to start TB treatment ranges from 0-50% depending on the severity of the presentation ^3^. Uniform case definitions of probable and possible TBM exist for research purposes, why not starting to use them in clinical care ^4^^,^^5^? Prediction rules including these case definitions allow for the calculation of the post-test probability and may help in taking the decision to treat presumptive TBM ^6^. They can be used in settings where an LP can be done.

**Theoretical framework of health systems**

 I fear that some bias may have crept in, because the author is a neurologist. As you mention yourself, TBM may not be seen as a public health priority by policy makers. Therefore we have to be careful to propose a theoretical framework and performance indicators that are realistic. I have personally never had access to a neurologist when working in Asia or Africa, and I still was able (I think) to treat patients with TB meningitis. In many settings referral of a patient to a tertiary care hospital will not be feasible or acceptable. General doctors, infectious diseases specialists, TB specialists, clinical officers and nurses can be trained to do an LP, and use the information in a diagnostic rule to reach a certain probability of TB meningitis leading to empiric or confirmed diagnosis.  

 Therefore, instead of the neurologist trained in neuro-infections I would propose availability of appropriate diagnostics (whatever level) and staff trained to use adapted guidelines for diagnosis and treatment at each level.

 In general, before proposing improvements of management of TBM, using a theoretical health systems framework, it would be good to reach consensus on the indicators to measure, taking into account a broader perspective than the tertiary care level. Which intervention across the cascade will have the highest impact?

I confirm that I have read this submission and believe that I have an appropriate level of expertise to confirm that it is of an acceptable scientific standard, however I have significant reservations, as outlined above.

## References

[1] : Evaluation of the 2007 WHO Guideline to Improve the Diagnosis of Tuberculosis in Ambulatory HIV-Positive Adults. *PLoS ONE*.2011;6(4) : 10.1371/journal.pone.0018502 10.1371/journal.pone.0018502 PMC307183721494694

[2] : Evaluation of the 2007 WHO guideline to diagnose smear negative tuberculosis in an urban hospital in Ethiopia. *BMC Infectious Diseases*.2013;13(1) : 10.1186/1471-2334-13-427 10.1186/1471-2334-13-427 24020936PMC3849989

[3] : Determining clinical decision thresholds for HIV-positive patients suspected of having tuberculosis. *Evidence Based Medicine*.2017;22(4) : 10.1136/ebmed-2017-110718 132-138 10.1136/ebmed-2017-110718 28716809

[4] : Standardized methods for enhanced quality and comparability of tuberculous meningitis studies. *Clinical Infectious Diseases*.2016; 10.1093/cid/ciw757 10.1093/cid/ciw757 28172588PMC5399942

[5] : Uniform Research Case Definition Criteria Differentiate Tuberculous and Bacterial Meningitis in Children. *Clinical Infectious Diseases*.2014;59(11) : 10.1093/cid/ciu665 1574-1578 10.1093/cid/ciu665 25139968

[6] : Xpert Ultra's place in the diagnosis of tuberculous meningitis. *The Lancet Infectious Diseases*.2018;18(3) : 10.1016/S1473-3099(18)30091-4 248-249 10.1016/S1473-3099(18)30091-4 29485086

# Response

**Published:** 2020-01-28

**Authors:** reinout van crevel

**Affiliations:** Radboud University Nijmegen Medical Centre, The Netherlands

Dear editor

 Please belowfind our point by point to the reviewer’s comments:

1. The cascade of care for TB meningitis, a hypothetical framework.  main problem here is how to determine the number of patients with TB meningitis in the community, your denominator? We know how many are diagnosed, but we have no idea on how many are unnoticed. How would you estimate the denominator?

**Answer:** *We think this will be impossible. It is a relatively rare disease, that is hard to diagnose and may start with non-specific symptoms. Barriers to health access and diagnosis may result in patient dying in the community, or with a false diagnosis; it is even hard to establish the number of patients **diagnosed** with TB meningitis.*

  

1. You mention that a problem of the cascade is that it only applies to an ideal scenario, and that it may not be available in all settings. I do not entirely agree, you may also discuss the appropriate level of care adapted to the available resources.

**Answer:** **This is a valid point. We now write** *: *It should be noted that this cascade is not based on universal international guidelines but rather represents an ‘ideal scenario’ that in high-burden settings may only exist in some centers, with a qualified or experienced team of professionals and appropriate services.

  

1. In the second loss of the cascade I would also add the **awareness** among health care workers to think about tuberculosis, as the initial step in the diagnostic process. Many patients with grade 1 BMRC severity of TBM (GCS normal and no focal signs) may never get a diagnostic work-up started.

**Answer:** **This is a valid point we now write**: Awareness among health care workers to think about brain infections and tuberculosis is needed, and an appropriate diagnostic workup has to be done

  

1. And at the end (part of the second loss), failure to make a diagnosis of **presumptive TB meningitis**, because all tests are negative. No test is sensitive enough to rule out TB meningitis, and clear guidance should be given to health care workers when to go for a presumptive TB diagnosis and treatment. In HIV-TB co-infected patients, the guidelines to improve the diagnosis of smear-negative TB in HIV prevalent settings (WHO 2006) led to a reduction in diagnostic delay and treatment delay ^1^ ^,^ ^2^.

**Answer: we now write** Unfortunately, no single diagnostic CSF can rule out TBM

 The required probability of TBM to start TB treatment ranges from 0-50% depending on the severity of the presentation ^3^. Uniform case definitions of probable and possible TBM exist for research purposes, why not starting to use them in clinical care ^4^ ^,^ ^5^? Prediction rules including these case definitions allow for the calculation of the post-test probability and may help in taking the decision to treat presumptive TBM ^6^. They can be used in settings where an LP can be done.

**Answer:** **We agree with the reviewer that a presumptive diagnosis should lead to tre3atment (we write: **After a presumptive or confirmed diagnosis of TBM is made, treatment should be started .. ), **and severity will dictate the level of uncertainty we are ready to accept (we have added **: , “..especially for critically ill patients”

**the international case definitions are not based on empiric data and have not been cross-validated between settings. This is a complex matter in the absence of a gold standard, and we feel this is a bit beyond the scope of this article.**

**Theoretical framework of health systems**

 I fear that some bias may have crept in, because the author is a neurologist. As you mention yourself, TBM may not be seen as a public health priority by policy makers. Therefore we have to be careful to propose a theoretical framework and performance indicators that are realistic. I have personally never had access to a neurologist when working in Asia or Africa, and I still was able (I think) to treat patients with TB meningitis. In many settings referral of a patient to a tertiary care hospital will not be feasible or acceptable. General doctors, infectious diseases specialists, TB specialists, clinical officers and nurses can be trained to do an LP, and use the information in a diagnostic rule to reach a certain probability of TB meningitis leading to empiric or confirmed diagnosis.  

 Therefore, instead of the neurologist trained in neuro-infections I would propose availability of appropriate diagnostics (whatever level) and staff trained to use adapted guidelines for diagnosis and treatment at each level.

**Answer: we have and replaced “neurologists” with “**doctors or other health staff”

 In general, before proposing improvements of management of TBM, using a theoretical health systems framework, it would be good to reach consensus on the indicators to measure, taking into account a broader perspective than the tertiary care level. Which intervention across the cascade will have the highest impact?

**Answer: we totally agree and already wrote: “.. **Based on such an assessment, interventions that will help most in a particular setting can be selected”

 We thank this reviewer for her helpful comments which we hope we have addressed appropriately.

 On behalf of our co-authors

 Reinout van Crevel

# Peer review: Reviewer response for version 1

**DOI:** 10.21956/wellcomeopenres.16982.r37228

**Published:** 2020-01-24

**Authors:** Irene Andia-Biraro

**Affiliations:** 1 Department of Internal Medicine, College of Health Sciences, Makerere University, Kampala, Uganda; 2 MRC/UVRI &LSHTM Uganda Research Unit, Entebbe, Uganda

The authors are reporting on a very important topic-tuberculous meningitis, a disease with high fatality. However, the review elaborates mainly on the factors affecting the cascade of care for TBM rather than proposing an actual cascade of care to guide clinicians. 

 It was also not clear how much patient factors such as stigma influenced the pre-admission and post discharge flow since TB related stigma is still one of the greatest barriers to TB control in the developing world.

 With regards to the proposed cascade in figure 1, what informed the decline rate across the 7 care points? Is the loss at a steady, equal rate? Also, why is y-axis labelled as survival and not frequency or number of patients?

I confirm that I have read this submission and believe that I have an appropriate level of expertise to confirm that it is of an acceptable scientific standard.
